# Student Perceptions of Motor, Mental and Social Benefits and the Impact of Practicing Recreational Figure Skating

**DOI:** 10.3390/bs8120110

**Published:** 2018-11-27

**Authors:** Anca Ionescu, Dana Badau

**Affiliations:** 1 Interdisciplinary Doctoaral School, Transilvania University of Brasov, Brasov 500036, Romania; ionescuanca12@gmail.com; 2 Department of Human Movement Sciences, University of Medicine, Pharmacy, Science and Technology of Targu Mures, Targu Mures 540139, Romania

**Keywords:** benefits, motor capacity, mental capacity, social capacity, students of physical education and sport, figure skating

## Abstract

The study aims to highlight the impact of practicing recreational figure skating by evaluating the relationship between the perceptions of motor, mental and social benefits of practicing figure skating and the frequency and duration allocated for this recreational activity. The study included 143 students of physical education and sport specialization. The questionnaire was designed to evaluate the students’ perception about the benefits of practicing recreational figure skating; it included 30 items divided in three parts: motor, mental and social benefits. Each of them contained 10 items to be assessed by students using the Likert scale, with 2 items related to the duration and frequency of practicing figure skating during recreational time. The results were processed using SPPS 24. The results were statistically significant at *p* < 0.05. The Cronbach’s Alpha coefficient of the questionnaire was α = 0.965, suggesting that the items had high internal consistency. Using the Likert scale, we found the following: concerning the high score 5 points—the motor capacity 62.9%, the mental capacity 49.7%, the social capacity 49.7%; and for a low score of 1 point—mental and social capacity 1.4%, motor capacity 0.7%. The effect size was medium for almost all items. No statistically significant correlations were found between the result of the questionnaire and the frequency and duration of practicing figure skating during free time. Figure skating is considered by students to be one of the activities through which a series of parameters of physical development and level of physical fitness can be improved through the expansion of motor skills. Also, the practice of figure skating contributes to the formation of proactive behaviors by improving the mental and social abilities of practitioners.

## 1. Introduction

The complex development of individuals in mental, physical and social aspects is the main objective of physical education [1,2,3]. Physical activity (PA) behavior is an important parameter of a lifestyle, being determined by a variety of personal, social, environmental factors, embodied in the emotional and instrumental concepts of PA, and also in social pressure and behavioral control in PA performance [4,5]. The concept of education is in a permanent state of change and modernization under the influence of pedagogical and social traditions and trends, requiring curricular adaptations in order to achieve the main educational objectives and the formation of complex specialized competences and behaviours. Physical education is an important component of general education, expressed using the motor activity aimed at optimizing the student’s biological and psychological potential, in order to increase the quality of life. Modernising the academic process by combining different methods and contexts is based on the exploration and experimentation of sporting and recreational activities that promote the growth of motor capacity, intellectual potential and social integration of students of physical education and sports field [4,5,6,7].

Physical education is one of the constantly evolving and adapting disciplines of education according to the new European-level standards. In this sense, the World Health Organization (WHO) published the European Physical Activity Strategy for WHO European Region 2016–2025, which proposes to ensure the adaptability of physical education programs in different contexts [8]. The need to change and adapt the content of the Physical Education discipline is essential in order to increase attractiveness in the context of a society that is increasingly inactive and reluctant to practice physical exercise [9,10,11,12,13]. Qualitative changes in physical education and sport occur when educators and teachers experienced directly and understood the implications of certain techniques and practices. Also, the education process is the foundation of positive behavior for the individual in society [14,15]. Regarding figure skating, the studies revealed that teachers’ skills in teaching this sporting branch are a decisive factor that influences the interest and the effect of skating on the motor, intellectual, psychological, and social capacities of pupils [16,17]. Figure skating is a complex sport that contains some specific features for the school level: a high level of physical abilities, artistic and aesthetic sense of movements, adaptation to the environment of the skating rink, good spatial orientation, etc. [18,19,20,21].

At the academic level, in Romania, figure skating was introduced as a compulsory discipline only in 2016, when Romanian National Academic Council of Academic Evaluation and Accreditation of High Education Institutions (ARACIS) standards were modified, and was included in the specialty discipline category [22]. According to ARACIS standards, figure skating is included in 2 categories of disciplines mandatory for the program of physical education and sports license: Theory and practice in expression sports (dance, folk dance, figure skating, synchronous swimming, artistic skiing) and in the practical winter sports disciplines performed in training camps (skiing, skating, hockey) [22]. The teaching methodology of figure skating requires appropriate knowledge and skills from teachers, in order to meet specific educational objectives. According to ARACIS standards, all curriculum subjects are divided into 3 categories: fundamental, specialized, and optional. Some of the sport disciplines can be categorized simultaneously into different categories depending on the specter of sport, but the option of including them in the curriculum respects the university autonomy. In this context, figure skating appears in the category of winter sports and in the category of expression sports. Each academic program is designed on the basis of university autonomy and the available local and regional infrastructure; therefore, each faculty can opt for the inclusion or exclusion of figure skating from the curriculum of students from the physical education and sports program. The analysis of the academic curricula specific for the faculties of physical education and sports in Romania revealed that figure skating is included in only two faculties as a discipline thought the country. Figure skating is a sport with great traditions in Europe and also across the world, having this in mind and also the specific of the Transilvania University faculty of PE which is Physical Education and Mountain Sports, we decided to undertake this study through which we want to highlight how students in the physical education and sport specialization perceive the role of figure skating and, at the same time, whether, on the basis of the specific knowledge and skills accumulated in the academic process, they will include this sport within the personal recreational activities. Although figure skating is an attractive sport, the technical and motor requirements that it involves makes this sport not often found among the youth’s recreational preferences in terms of the benefits of the study [23,24]. At an international level, research shows an increase in the number of recreational practitioners, which is in contradiction with the current trend in Romania, despite the continuous development of infrastructure. Considering the history of practicing this sport in relation to the present trend for Romania, we observe a decrease in both the number of practitioners and also the frequency of practicing as a competitive sport and as a recreational activity. This contradiction forced us to identify the main positive and negative, determinant and specific aspects. Students from Physical Education and Sports as future specialists of PA should be primarily responsible for promoting a diversified range of recreational sports activities. The context of approaching this theme is a complex one regarding the psychological, motor and practical aspects of the figure skating practice. Through the results recorded in this study, we want to convey new evidence of the role of figure skating and the impact of practicing as recreational leisure activity. Identifying figure skating specific driving patterns can influence the formation of proactive behaviors and the expansion of motor skills. Identifying specific mental aspects of this sport will allow educators to focus on the formation of correct and timely mentalities and overcome mental barriers determined by the specific risks of this sport. From a social point of view, the hierarchy of the role and benefits of figure skating practice will hopefully lead to the formation of fair behaviors and collectivity behavior of fair play in skating rinks. Studying the impact of practicing recreational figure skating among students that would eventually become the teachers that will promote the sport with young children is of tremendous importance because studies have shown that if the teachers are familiar with the sport that they will be teaching they can increase the attractiveness for pupils [25,26,27]. The literature review revealed insufficient evidence related to the appreciation of the benefits of practicing figure skating by students from the physical education and sports specialization who will become future school promoters of this sport. In this context, we believe that our study will bring new evidence regarding students’ perceptions about the benefits of implementing figure skating in the academic and school levels. Current and modern trends of academic curricula are focused on improving the professional training of future physical education teachers in line with the specific standards [27,28,29,30].

In this respect, the first aim of our study was to assess students perceptions about the impact of figure skating as a recreational activity in order to improve the motor, mental and social capacities through implementation of the artistic skating discipline in the academic curriculum of physical education and sports specialization. The second aim was to evaluate the correlation between students’ perceptions of benefits of practicing figure skating as a recreational activity and the frequency and duration of training allocated for this activity, at end of the course, during free time.

## 2. Materials and Methods

### 2.1. Study Design

The research was a study conducted between December 2017 and April 2018. Within our study, we applied one questionnaire for students’ perception about the benefits of practicing figure skating on motor capacity, physical capacity and social integration. The questionnaires were developed with Google Forms and were delivered directly to the students at the end of the course, after the final exam, under the supervision of the coordinating teachers. Participation in the study was voluntary. The participants are all students from the PE Faculty and their theoretical knowledge includes aspects about the technical elements of figure skating, the risks of injury the process of arbitration and also about the rules and regulations provided by the International Skating Union. During this practical course, students were taught basic figure skating elements such as three turns, waltz jump, forward and backwards stroking and the basic spins and jumps. The course was conducted over a single semester, in the first year of study, including a total of 14 h of practical work.

### 2.2. Participants

The study sample included 143 students. This cross-sectional study included students of Faculty of Physical Education and Mountain Sports at Transilvania University of Brasov. The Faculty of Physical Education and Mountain Sports from Brasov is the only faculty in Romania specialized in winter sports: skiing, ice skating, hockey. To meet the inclusion criteria, students had to be active and healthy with good attendance (80%) of the figure skating course. The exclusion criteria: less than 80% attendance of the course, incorrect completion of the questionnaire. Age sample characteristics: 21.57 ± 1.09 age of years; male group 79 (55.2%), female group 64 (44.8%). Students had no experience in figure skating before starting the specific curriculum for the specialization of physical education and sports. The course was conducted over a single semester, in the first year of study, including a total of 14 h of practical work. Study participants are active athletes who practice mostly team sports, 62%, and individual sports, 38%, but none is a practitioner of figure skating.

### 2.3. Measures

For this study, we have created one questionnaire, called The Questionnaire for benefits of practicing recreational figure skating, containing 30 items divided in 3 parts depending on the capacity that is intended to be improved. Its three parts aim at the following capacities: motor, mental and social. Each part includes 10 items that correspond to the main specific objectives. The 10 objectives analyzed for each of the three plans: motor, mental and social, were selected together with the experts from the Educational Science and Physical Education of the Transilvania University of Brasov, Romania. The questionnaire was designed with a 5-point Likert scale; 1 representing the minimum level, and 5 the highest level of appreciation by the students of the physical education and sports program. The items of each part were selected according to the physical, mental and social motivations of figure skating identified in the specialized literature of the Romanian Skating Federation [31]. The online form included 2 questions about the frequency and duration of skating during leisure time after the course. Frequency was estimated with the Likert scale: 1 point representing never, 5 points every day. The duration of the skating per training session during leisure time had the following Likert scale: 1 point 30 min, 2 points 60 min, 3 points 90 min, 4 points 120 min, 5 points 150 min.

### 2.4. Statistical Analysis

The data were processed by using IBM-SPSS 24. The statistical analysis included the arithmetic average (X), standard deviation (SD), Student’s *t* test (*t*), Pearson’s (r) and Spearman’s correlations (r_s_), and effect size (d). Effect size was determined according to the Cohen scale [32]. The Pearson index was chosen to highlight the correlation between parts of the questionnaire for the benefits of practicing recreational figure skating in terms of motor, mental and social capacities. The PCA for the data included: the Bartlett’s sphericity test and the Kaiser-Meyer-Olkin (KMO). The reliability, or the internal consistency, of the questionnaire was calculated using the Cronbach’s alpha statistical index (α). Descriptive statistics (mean ± SD) were calculated for all variables. The value of statistical significance was set at *p* < 0.05. The Rotation Method, Varimax with Kaiser Normalization was used to identify the important factors. All 30 items of the questionnaire were analyzed. Only items which had positive values higher than 0.6 on one factor that contributed directly to this pattern were taken into consideration. A high score of the items for a pattern indicated a high educational relevance.

## 3. Results

### 3.1. The Part of the Questionnaire about the Benefits of Figure Skating for Motor Capacity

Cronbach’s Alpha for the 10 items of the part of the questionnaire of the benefits of figure skating on motor capacity was α = 0.922, suggesting that the items had high internal consistency. The Keiser-Meyer-Olkin measure verified the sampling adequacy for analysis of the questionnaire about the impact of figure skating on motor capacity, KMO = 0.915, which is above Keiser’s criterion (> 0.5). Bartlett’s test of sphericity χ^2^ (45) = 882.226, *p* < 0.00, indicated that the correlation between items was sufficiently high for PCA. For the motor part of the questionnaire of the benefits of figure skating, the values were statistically significant for *p* < 0.05, thus X ± SD was 4.493 ± 0.098, *t* (df9) 144.171, *p* < 0.00. Statistical processing of Student’s t test highlighted statistically significant results for *p* < 0.05 (Table 1). The study revealed a small size effect for all items, with 2 exceptions that had a medium effect size for items: 1. General and harmonious physical development and 2. Increasing the effort capacity (Table 1). The average number of the subjects (and percentage) according to the points of the Likert scale for the part of the questionnaire regarding the impact of figure skating on motor capacity was as follows: for maximum 5 points, 90 students (62.9%); for 4 points, 35 students (24.5%); for 3 points, 16 students (11.2%); for 2 points and 1 point, only 1 student (0.7%), (Table 1). The results highlight a high motor appreciation of practicing figure skating for 98.6% of students that gave more than 3 points. However, 1.4% of subjects considered that the contribution of practicing figure skating did not sufficiently influence their motor skills, probably because they considered the sport to be more difficult, with a certain degree of risk and not a part of their preferences. The analysis of the average results permits a hierarchy of the impact that recreational figure skating has on the main motor parameters. Thus, in the opinion of respondents, figure skating develops mainly coordination skills (item 10), improving muscle toning (item 3) and proprioception (item 9). The weakest results were obtained from items 1, 2, 4, 8 which focused on general and harmonious physical development, improving the capacity of the body, improving psychomotor capacity and improving motor skills. The results reveal that respondents consider mainly the role of coordination and psychomotority and less the improvement of the motor skills and the functional capacity of the body (Table 1).

### 3.2. The Part of the Questionnaire about the Benefits of Figure Skating on Mental Capacity

High internal consistency has been found for the 10 items of the part of questionnaire about the impact of figure skating on mental capacity, Cronbach’s Alpha was α = 0.930. For analysis of the questionnaire part about the impact of figure skating on mental capacity, the Keiser-Meyer-Olkin measure verified the sampling adequacy, KMO = 0.907, which is above Keiser’s criterion *p* > 0.5. The correlation between items was sufficiently large for PCA related to Bartlett’s test of sphericity χ2 (45) = 957.254, *p* < 0.00. For the mental capacity part of the questionnaire of the benefits of figure skating, the values were statistically significant for *p* < 0.05, thus X ± SD was 4.235 ± 0.110, *t* (df9) 121.553, *p* < 0.00. The normality of the results regarding the impact of figure skating on mental capacity is highlighted by the *t*-test values and Z values that were statistically significant for *p* < 0.05 (Table 2). The study highlighted a medium size effect for all items with exceptions for item 4. Improving attention and memory and item 8. Improving self-esteem, which had a large effect (Table 2).

The average number of the subjects (and percent) according to the points of the Likert scale for the part of the questionnaire about the benefits of figure skating on mental capacity were: for maximum 5 points, 71 students (49.7%); for 4 points, 40 students (28%); for 3 points, 26 students (18.2%); for 2 points, 4 students (2.8%); for 1 point, 2 students (1.4%), (Table 2). Figure skating has a significant contribution to the development of mental capacity reflected by the high percentage (95.9%) of the appreciation of its benefits listed in our study with 3–5 points. Appreciation with 1–2 points in a percentage of only 4.1% was considered to be the result of the risks that figure skating can cause as a result of the high incidence of injury. Respondents considered that recreational figure skating has a positive influence in particular for the following items: the role of education (item 5), the formation of proactive behaviors (item 6) and the improvement of body image (item 3). The least influenced components of mental parameters are: reducing anxiety (item 7), stimulating creativity (item 9) and increased adaptability to different environments (item 10). We would like to highlight the fact that respondents appreciate especially the formative and educational components of mentality formation and proactive behaviors (Table 2).

### 3.3. The Part of the Questionnaire about the Benefits of Figure Skating on Social Capacity

Cronbach’s Alpha for the 10 items of the part of the questionnaire of benefits of figure skating on social implication capacity was α = 0.931, suggesting that the items had high internal consistency. The Keiser-Meyer-Olkin measure verified the sampling adequacy for analysis of the questionnaire part about the benefits of figure skating on social implication capacity, KMO = 0.912, which is above Keiser’s criterion (>0.5). Bartlett’s test of sphericity χ^2^ (45) = 1014.721, *p* < 0.00, indicated that the correlation between items was sufficiently large for PCA. For the social part of the questionnaire of the benefits of figure skating, the values were statistically significant at *p* < 0.05, thus X ± SD was 4.233 ± 0.151, *t* (df9) 88.433, *p* < 0.00. We found a small size effect for items: 4. Behavior self-regulation, 5. Improving communication and 8. Developing independence in actions and relations. Medium effect sizes were found for all other items (Table 3).

According to Table 3, for each item of this part of the questionnaire, the normality of the results highlighted by the Z values and t-value were statistically significant for *p* < 0.05. The average number of the subjects (and percent) according to the points of the Likert scale for the part of the questionnaire regarding the impact of figure skating on social implication capacity were: for maximum 5 points, 70 students (49%); for 4 points, 43 students (30.1%); for 3 points, 23 students (16.1%); for 2 points, 5 students (3.5%); for 1 point, 2 students (1.4%), (Table 3). Despite the positive appreciation of social-induced skating, however, 4.9% of the study subjects gave small scores (1–2 points). We consider that this reflects a certain mentality that does not include this sport in the top of the Romanian sports preferences, which are focused especially on the trending sports, namely, on team sports, aquatic sports and alpine skiing (Table 3). From the perspective of social integration, respondents considered recreational figure skating to contribute to the development of the spirit of competitiveness (item 2), developing a proactive lifestyle (7) and increasing the quality of life (3). Students identified that recreational figure skating has the least impact manifested in the following items: Improving communication (item 5), the possibility of multidirectional education (item 9) and social integration (item 10). The results are surprising because the determined parameters of social integration and socialization are not considered to be developed through figure skating, probably due to the specific risk factors and the way of organizing the activity and the poorly socialized sport mentality (Table 3).

### 3.4. The Questionnaire about the Benefits of Figure Skating

Cronbach’s Alpha for the 30 items of the questionnaire was α = 0.965, suggesting that the items had high internal consistency. The KMO measure verified the sampling adequacy for analysis of the questionnaire part about the impact of figure skating on social implication capacity, KMO = 0.927, which is above Keiser’s criterion (>0.5). Bartlett’s test of sphericity χ^2^ (435) = 3525.693, *p* < 0.00, indicated that the correlation between items was sufficiently large for PCA. For the whole questionnaire the values were statistically significant for *p* < 0.05, thus X ± SD was 4.320 ± 0.171, *t* (df29) 138.211, *p* < 0.00.

The results of the study highlight the following hierarchy of the appreciation of the subjects of the study in relation to the benefits of figure skating in the three capacities concerned: 5 points—the motor capacity 62.9%, the mental capacity 49.7%, the social capacity 49.7%; 4 points—mental capacity 30.1%, social capacity 28%, motor capacity 24.5%; 3 points—social capacity 18.2%, mental capacity 16.1%, motor capacity 1.2%; 2 points—social capacity 3.5%, mental capacity 2.8%, motor capacity 0.7%; 1 points—mental and social capacity 1.4%, motivation capacity 0.7%.

An initial analysis was run to obtain eigenvalues for each component of the results of our study. Four components had eigenvalues over Keiser’s criterion of 1 and in combination explained 67.147% of the variance (Table 4). Rotation method trough the Varimax with Kaiser Normalization converged in 8 iterations. The primary factor loadings over 0.6 are in bold font. The components were renamed according to the higher value obtained on the vertical factor: F1 The possibility of multidirectional education; F2 Improving motor skills; F3 Mental relaxation; F4 Developing the spirit of competitiveness (Table 4). The first component can be defined as a socially determined component in terms of the values of the first three items determined, namely: the possibility of multidirectional education (item S9), increasing independence in actions and relations (item S8) and increasing active and participative involvement (item S6). The second component can be defined as a driving determinant component in terms of the values of the first three items, namely: improving motor skills (item M8), improving mobility (item M7) and improving proprioception (item M9). The third component can be defined as a mental determinant component in terms of the values of the first 3 items determined: mental relaxation (item P1), fighting stress (item P2) and educational role (item P5). The fourth component can be defined by the only parameter that complied with the benchmark criterion, namely item S2 Developing the spirit of competitiveness (Table 4).

Analysis of the Spearman correlation between all parts of the questionnaire of benefits of figure skating (Table 5) reveals positive correlation between all parts: motoric, mental and social. All correlations were statistically significant (Table 5). Analysis of the correlation between the motor, mental and social parts of the questionnaire of benefits of figure skating (Table 5) reveals positive correlation between motor and mental parts, and negative correlation between motor and social parts and between social and mental parts. All correlations were statistically significant, with only one exception between mental and motor parts of the questionnaire (Table 5).

The results were statistically significant at the 0.05 level, and the effect sizes were medium (Table 6).

We found no significant correlation between duration and frequency of practicing figure skating as a recreational activity. In the same time, the study revealed a medium correlation between duration and frequency of practicing figure skating during free time; the correlations were statistically significant (Table 7). We have identified an insignificant correlation between questionnaire results and the duration and frequency of practicing figure skating in leisure time that could have multiple intrinsic or extrinsic motivations.

## 4. Discussion

The results of the study have allowed us to achieve a hierarchy of the social, mental and physical benefits of practicing recreational skating among students from the physical education specialization as future PA specialists, which will optimize mentalities and behaviors in promoting this sport. Figure skating has many educational valences; the results of our study facilitate a hierarchy of the benefits of this sport in terms of the main parameters of motor, mental and social capacity within the academic activities specific to physical education and sports programs. The results of the study for the three parts (motor, mental and social) for the questionnaire of the benefits of figure skating highlight the higher percentages obtained for all items for the maximum answer 5. The best recorded results regarding the impact of the figure skating on the motor capacity were recorded by the following items: item 10. Development of the coordination qualities; item 9. Improving proprioception and item 3. Improving muscle toning. The benefits of figure skating on mental capacity have best been appreciated in the following items: item 5. Educational role; item 6. Forming proactive behaviors; item 8. Improve self-esteem. Students participating in the study considered that figure skating influences most of the following 3 social parameters: item 2. Development of competitiveness spirit; item 7. Forming a proactive lifestyle; item 3. Increasing the quality of life. The effect size was medium for a large number of items of the questionnaire and that reflected the benefits of practicing the figure skating in terms of motor, mental and social plans of lifestyle.

The results of the study confirmed only in part the conclusions of previous studies that address the benefits of different physical activities within the academic level. Despite identifying the major benefits of this activity, subjects do not include skating among their recreational preferences, probably because of the high degree of difficulty, risk, and insufficiently oriented mentality and behavior towards practicing different activities and in different environments. Studies have shown that experimenting with ways to improve student formative activity through curricular diversification creates favorable conditions for training attitudes and extending professional skills to future physicists [33,34]. Examination of the field literature shows that there are a number of studies [35,36,37,38] carried out in this country and abroad that show parallels with the findings of this study. In this way, the study revealed the positive impact of different specific activities of physical education on the psychological and mental levels of students [39], on the social integration and relationships level of students [40,41,42] and on the physical fitness [19,43,44,45]. According to studies, approximately 54–57% of the total daily hours are used by adults for sedentary activities [46,47], and the prevalence of the same type of behavior is significantly higher for the student [48]. The motivations for practicing various sports and physical exercises are varied, such as intrinsic or extrinsic nature, focused on self-image, self-esteem, and stress management [48]. Comparing figure skating with other winter sports found in the top of the recreational preferences of PE students, namely, alpine skiing, snowboarding, cross-country skiing, figure skating is one of the sports that develops: expressivity, artistic sense, coordination, etc. [49,50,51,52]. Results of the study showed that despite identifying by respondents the benefic influence that figure skating has, practice is still insufficient.

## 5. Limitations

The study was aimed only at students from the Faculty of Physical Education and Sports in Brasov and it should be extended to other professional categories that are involved in the teaching of figure skating for recreational purposes. Assessing the benefits of figure skating on the components of motor, mental and social capacity through the questionnaire can be of limited value. Strengths. The study has allowed an analysis of how students in the physical education and sports program appreciate the benefits of figure skating on motor, mental and social capacity. The results of the study allowed us to achieve a hierarchy of the benefits of figure skating on the main parameters of motor, mental and social capacity. Future studies should focus on highlighting the level of theoretical expertise in sports and physical skills as well as on health impacts by practicing skating for recreational purposes at school and academia. Researchers in the field of physical activity should be directed to identify attractive and optimal ways of practicing physical activities in relation to new educational tendencies, preferences of practitioners, social requirements, etc. The current contexts of education that will apply in the future require complex interdisciplinary approaches.

## 6. Conclusions

The results of the study highlighted the major benefits on the motor, mental and social capacities of figure skating in the view of students from the physical education and sports program. Figure skating is considered by the students to be one of the activities through which a series of parameters of physical development, and level of physical fitness can be improved through the expansion of motor skills. Also, the practice of figure skating contributes to the formation of proactive behaviors by improving the mental and social abilities of practitioners. In spite of the positive appreciation of the benefits of skating practice highlighted in our study, we have found a lack of inclusion of this sport in recreational leisure activities. The artistic, motor and technical characteristics of figure skating, combined with the risks of injury and the characteristic of ice and skating, determine the development of specific motor skills to improve motor control, the ability to take risks and overcome mental barriers, developing courage, self-esteem and implicitly adjusting the behavior of adapting the exercise to the community in a space delimited and influenced by the characteristics of the ice. The lack of participants is due to a certain mentality focusing on the trend of practicing predominantly more popular sports in Romania.

The implementation of figure skating in the academic curriculum of the physical education and sports program is a sure premise in order to expand the skills and competences of the students from the physical education and sports program. By developing the competences of the students from the physical education and sports program, corroborated with the perspective of future professional activities as teachers, figure skating can be optimally implemented in the content of the physical education subject to school educational levels (primary, gymnasium and high school).

## Figures and Tables

**Table 1:** Descriptive statistics of the motor part of the questionnaire of benefits of practicing recreational figure skating.

Items	Responses Weight N (%)					Statistical Data			
	5 Points	4 Points	3 Points	2 Points	1 Point	X	SD	*t*	d
1. General and harmonious physical development	77 (53.8)	41 (28.7)	22 (15.4)	2 (1.4)	1 (0.7)	4.33	0.838	61.82	0.61
2. Increasing the effort capacity	74 (51.7)	44 (30.8)	23 (16.1)	1 (0.7)	1 (0.7)	4.32	0.818	63.12	0.58
3. Improving muscle toning	99 (69.2)	31 (21.7)	12 (8.4)	1 (0.7)		4.59	0.673	81.53	0.22
4. Improving psychomotor capacity	89 (62.2)	36 (25.2)	17 (11.9)	-	1 (0.7)	4.48	0.758	70.66	0.41
5. Improving body posture	88 (61.5)	39 (27.3)	15 (10.5)	1 (0.7)	-	4.49	0.710	75.64	0.32
6. Developing conditional motor skills	87 (60.8)	41 (28.7)	13 (9.1)	2 (1.4)	-	4.49	0.720	74.50	0.34
7. Improving mobility	95 (66.4)	30 (21.0)	18 (12.6)	-	-	4.53	0.709	76.46	0.30
8. Improving motor skills	87 (60.8)	38 (26.6)	18 (12.6)	-	-	4.48	0.710	75.43	0.32
9. Improving proprioception	100 (69.9)	26 (18.2)	16 (11.2)	1 (0.7)	-	4.57	0.716	76.29	0.31
10. Developing coordination skills	104 (72.7)	25 (17.5)	13 (9.1)	1 (0.7)	-	4.62	0.679	81.32	0.22

X—average, SD—standard deviation, N—number of students, *t*—Student’s *t* test value, d—effect size.

**Table 2:** Descriptive statistical of the mental part of the questionnaire of the benefits of practicing recreation figure skating.

Items	Responses Weight N (%)					Statistical Data			
	5 Points	4 Points	3 Points	2 Points	1 Point	X	SD	*t*	d
1. Mental relaxation	69 (48.3)	42 (29.4)	27 (18.9)	5 (3.5)	-	4.223	0.875	57.693	0.73
2. Fighting stress	70 (49.0)	39 (27.3)	28 (19.6)	5 (3.5)	1 (0.7)	4.202	0.923	54.418	0.58
3. Improving body image	74 (51.7)	42 (29.4)	23 (16.1)	3 (2.1)	1 (0.7)	4.293	0.862	59.522	0.68
4. Improving attention and memory	72 (50.3)	38 (26.6)	26 (18.2)	7 (4.9)	-	4.223	0.914	55.212	0.81
5. Educational role	84 (58.7)	40 (28.0)	17 (11.9)	1 (0.7)	1 (0.7)	4.433	0.783	67.688	0.40
6. Forming proactive behaviors	80 (55.9)	37 (25.9)	22 (15.4)	3 (2.1)	1 (0.7)	4.342	0.864	60.046	0.64
7. Reducing anxiety	64 (44.8)	38 (26.6)	33 (23.1)	6 (4.2)	2 (1.4)	4.090	0.985	49.657	0.52
8. Improving self-esteem	75 (52.4)	44 (30.8)	21 (14.7)	1 (0.7)	2 (1.4)	4.321	0.852	60.620	0.91
9. Stimulating creativity	60 (42.0)	47 (32.9)	30 (21.0)	3 (2.1)	3 (2.1)	4.104	0.947	51.818	0.47
10. Increased adaptability to different environments	66 (46.2)	39 (27.3)	31 (21.7)	6 (4.2)	1 (0.7)	4.139	0.946	52.306	0.66

X—average, SD—standard deviation, N—number of students, *t*—Student’s *t* test value, d—effect size.

**Table 3:** Descriptive statistical of the social part of the questionnaire of benefits of practicing recreational figure skating.

Items	Responses Weight N (%)					Statistical Data			
	5 Points	4 Points	3 Points	2 Points	1 Point	X	SD	*t*	d
1. Socializing	60 (42.0)	51 (35.7)	26 (18.2)	6 (4.2)	-	4.153	0.866	57.326	0.74
2. Developing the spirit of competitiveness	96 (67.1)	37 (25.9)	8 (5.6)	1 (0.7)	1 (0.7)	4.580	0.696	78.676	0.57
3. Increasing the quality of life	75 (52.4)	44 (30.8)	19 (13.3)	2 (1.4)	3 (2.1)	4.300	0.904	56.884	0.62
4. Behavior self-regulation	69 (48.3)	52 (36.4)	16 (11.2)	5 (3.5)	1 (0.7)	4.279	0.850	60.146	0.21
5. Improving communication	64 (44.8)	43 (30.1)	23 (16.1)	10 (7.0)	3 (2.1)	4.083	1.037	47.049	0.42
6. Increasing active and participative involvement	66 (46.2)	43 (30.1)	27 (18.9)	7 (4.9)	-	4.174	0.906	55.087	0.74
7. Developing a proactive lifestyle	77 (53.8)	45 (31.5)	17 (11.9)	4 (2.8)	-	4.363	0.800	65.151	0.79
8. Developing independence in actions and relations	68 (47.6)	39 (27.3)	28 (19.6)	7 (4.9)	1 (0.7)	4.160	0.954	52.143	0.34
9. The possibility of multidirectional education	66 (46.2)	35 (24.5)	36 (25.2)	5 (3.5)	1 (0.7)	4.118	0.953	51.681	0.69
10. Social integration	65 (45.5)	41 (28.7)	30 (21.0)	5 (3.5)	2 (1.4)	4.132	0.958	51.558	0.76

X—average, SD—standard deviation, N—number of students, *t*—Student’s *t* test value, d—effect size.

**Table 4:** Statistical Rotated Component Matrix of the questionnaire of the benefits of figure skating.

Items	Components			
	1	2	3	4
S9 The possibility of multidirectional education	**0.818**	0.312	0.139	0.132
S8 Developing independence in actions and relations	**0.799**	0.275	0.081	0.190
S6 Increasing active and participative involvement	**0.785**	0.195	0.196	0.261
S7 Developing a proactive lifestyle	**0.761**	0.256	0.030	0.193
S5 Improving communication	**0.753**	0.141	0.272	0.071
S10 Social integration	**0.734**	0.096	0.299	0.122
P8 Improving self-esteem	**0.714**	0.312	0.316	0.082
P10 Increased adaptability to different environments	**0.701**	0.338	0.185	−0.010
S4 Behavior self-regulation	**0.688**	0.251	0.169	0.390
S1 Socializing	**0.617**	0.134	0.265	−0.006
P4 Improving attention and memory	0.592	0.408	0.477	−0.060
P6 Forming proactive behaviors	0.584	0.334	0.255	−0.124
S3 Increasing the quality of life	0.565	0.258	0.421	0.373
P7 Reducing anxiety	0.564	0.464	0.417	−0.201
P9 Stimulating creativity	0.497	0.443	0.213	0.329
M8 Improving motor skills	0.202	**0.811**	0.083	0.071
M7 Improving mobility	0.342	**0.769**	0.056	0.070
M9 Improving proprioception	0.118	**0.724**	0.124	0.439
M3 Improving muscle toning	0.110	**0.723**	0.166	0.346
M10 Developing coordination motor skills	0.206	**0.705**	0.011	0.378
M5 Improving body posture	0.318	**0.677**	0.162	0.010
M2 Increasing the effort capacity	0.271	**0.659**	0.255	0.082
M4 Improving psychomotor capacity	0.344	**0.658**	0.311	−0.058
M6 Developing conditional motor skills	0.359	**0.634**	0.178	0.159
M1 General and harmonious physical development	0.111	0.596	0.308	−0.078
P1 Mental relaxation	0.258	0.182	**0.787**	0.233
P2 Fighting stress	0.351	0.188	**0.755**	0.256
P5 Educational role	0.488	0.380	**0.600**	−0.027
P3 Improving body image	0.472	0.341	0.505	0.094
S2 Developing the spirit of competitiveness	0.210	0.218	0.201	**0.707**
Eigenvalues	15.139	2.544	1.265	1.196
% of Variance	50.463	8.480	4.218	3.986

**Table 5:** Statistical correlation between the parts of the questionnaire about the benefits of practicing recreational figure skating.

Parameters		Motoric Part of QBRFS	Mental Part of QBRFS	Social Part of QBRFS
Motoric part of QBRFS	r/r_s_	1	0.377/0.530	−0.770/−0.610
	p_r_/p_rs_	-	0.141/0.021 *	0.015 */0.038 *
Mental part of QBRFS	r/r_s_	0.377/0.530	1	−0.748/0.698
	p_r_/p_rs_	0.141/0.021 *	-	0.020 */0.027 *
Social part of QBRFS	r/r_s_	−0.770/−0.610	−0.748/−0.698	1
	p_r_/p_rs_	0.015 */0.038 *	0.020 */0.027 *	-

QEIFS—the questionnaire of benefits of recreational figure skating, r—Pearson’s correlation, r_s_—Spearman’s correlation, p_r_—significant level of Pearson’s correlation index, p_rs_—significant level of Spearman’s correlation index, * Correlation is significant at the 0.05 level.

**Table 6:** Descriptive analyze of QBRFS and duration of practicing figure skating during recreational time.

Parameters	X	SD	*t*	d
QBRFS	4.320	0.171	138.211	0.59
Frequency	2.069	1.045	23.669	0.42
Duration	1.909	1.074	21.255	0.46

QBRFS—the questionnaire of benefits of recreational figure skating, X—average, SD—standard deviation, *t*—Student’s *t* test value, d—effect size.

**Table 7:** Statistical correlation between the questionnaire for benefits of recreational figure skating and frequency, and duration of practicing figure skating during recreational time.

		QBRFS	Frequency	Duration
QBRFS	r/r_s_	1	0.238/0.207	0.121/0.120
	p_r_/p_rs_		0.103/0.271	0.262/0.529
Frequency	r/r_s_	0.238/0.207	1	0.526 **/0.449 **
	p_r_/p_rs_	0.103/0.271		0.000/0.000
Duration	r/r_s_	0.121/0.120	0.526 **/0.449 **	1
	p_r_/p_rs_	0.262/0.529	0.000/0.000	

QBRFS—the questionnaire of benefits of practicing recreational figure skating, r—Pearson’s correlation. r_s_—Spearman’s correlation, p_r_—significant level of Pearson’s correlation index, p_rs_—significant level of Spearman’s correlation index, ** Correlation is significant at the 0.01 level.

## References

[1] Villardón-Gallego L., García-Carrión R., Yáñez-Marquina L., Estévez A. (2018). Impact of the interactive learning environments in children’s prosocial behavior. Sustainability.

[2] Ennis C.D. (2017). Educating Students for a Lifetime of Physical Activity: Enhancing Mindfulness, Motivation, and Meaning. Res. Q. Exerc. Sport.

[3] Mocanu G.D. (2018). Loisir/Activitati Motore de Timp Liber.

[4] Pate R.R., Berrigan D., Buchner D.M. (2018). Actions to Improve Physical Activity Surveillance in the United States.

[5] Blanchard C., Fisher J., Sparling P., Nehl E., Rhodes R., Courneya K., Baker F. (2008). Understanding physical activity behavior in African American and Caucasian college students: An application of the theory of planned behavior. J. Am. Coll. Health.

[6] Ene-Voiculescu C., Ene-Voiculescu V. (2015). The impact of outdoor play activities in school children. Sci. Bull. “Mircea Batran” Naval Acad..

[7] Badau A. (2017). Study of somatic, motor and functional effects of practicing initiation programs in water gymnastics and swimming by students of physical education and sports. Phys. Educ. Stud..

[8] The World Health Organization. http://www.euro.who.int/__data/assets/pdf_file/0010/282961/65wd09e_PhysicalActivityStrategy_150474.pd.

[9] Gorucu A., Tokay B., Badau A. (2017). The effects of three different type of exercises on aerobic and anaerobic power. Phys. Educ. Stud..

[10] Moldovan E., Enoiu R.S. (2012). Optimizing the practice of montainous tourism from the winter sports perspective. Bull. Transilv. Univ. Brasov Ser. IX Sci. Hum. Kinet..

[11] Cojocaru A.M., Cojocaru M. (2014). Study on power development in children adolescents in human motority. Appl. Mech. Mater..

[12] Allender S., Cowburn G., Foste C. (2006). Understanding participation in sport and physical activity among children and adults: A review of qualitative studies. Health Educ. Res..

[13] Chief Medical Officer (2004). At Least Five a Week: Evidence on the Impact of Physical Activity and Its Relationship to Health: A Report from the Chief Medical Officer.

[14] Rink J. (2014). Teaching Physical Education for Learning.

[15] Badau A. (2016). The level evaluation of motor qualities at medicine students. Ann. “Dunarea De Jos” Univ. Galati.

[16] Fromel K., Kudlacek M., Groffik D., Svozil Z., Simunek A., Garbaciak W. (2017). Promoting Healthy Lifestyle and Well-Being in Adolescents through Outdoor Physical Activity. Int. J. Environ. Res. Public Health.

[17] Liang Z. (2007). My Thoughts on Advancing the Teachers’ Skills in Skating Class. CNKI J..

[18] Slater L.V., Vriner M., Zapalo P., Arbour K., Hart J.M. (2016). Difference in agility, strength and flexibility in competitive figure skaters based on level of expertise and skating discipline. J. Strength Cond. Res..

[19] Radman I., Ruzic L., Padovan V., Cigrovski V., Podnar H. (2016). Reliability and Validity of the Inline Skating Skill Test. J. Sports Sci. Med..

[20] Lin C. (2011). Teaching reform and ports injury precaution in the skating curriculum in universities. Education and Educational Technology.

[21] Arapovic M., Sebic L. Relations of basic motor skills and stylized move structures of figure skaying. Proceedings of the 6th International Scientific Conference on Kinesiology: Integrative Power on Kinesiology.

[22] Standarde Specifice ARACIS, Comisiei de Specialitate: Educație Fizică si Sport. http://www.aracis.ro/fileadmin/ARACIS/Comunicate_Media/2016/Standarde_specifice_consultare/8._Standarde_ARACIS_-_Comisia_8._Arte_BEX.pdf.

[23] Eun J.J. (2014). Exploring the motivation motivation of figure skating participants as leisure sports. Korean J. Wellness.

[24] Alexadris K., Kouthouris C., Girgolas G. (2017). Investigating the relationships among motivation, negotiation, and alpine skiing participation. J. Leis. Res..

[25] Gabriel K.K.P., Morrow J.R., Woolsey A.L.T. (2012). Framework for physical activity as a complex and multidimensional behavior. J. Phys. Act. Health.

[26] Mattson J.M., Richards J. (2013). Early Specialization in Youth Sport. A Biomechanical Perspective. J. Phys. Educ. Recreation Dance.

[27] Wiersma L.D. (2000). Risks and benefits of youth sport specialization: Perspectives and recommendations. Pediatr. Exerc. Sci..

[28] Shi-Ping G.U.O. (2011). Safety Strategies in the Curriculum of Winter Ice Sports in Northern Universities. China Winter Sports.

[29] Ying L.I. (2012). Research on the Problems about Teaching Effect on the Ice in Universities. China Winter Sports.

[30] Jia H., Yang X. (2012). College Speed Skating Network Curriculum Implementation. Softw. Eng. Knowl. Eng. Theory Pract..

[31] Romanian Federation of Skating. http://www.frponline.ro/rezultateonline.html.

[32] Cohen J. (1988). Statistical Power Analysis for the Behavioral Sciencies.

[33] Iermakov S.S., Cieślicka M., Muszkieta R. (2015). Physical culture in life of Eastern European region students: Modern state and prospects of development. Phys. Educ. Stud..

[34] Marinho A., Mari dos Santos P., Manfroi M.N., Figueiredo J.P., Zeilmann Brasil B. (2017). Reflections about outdoor adventure sports and professional competencies of physical education students. J. Adventure Educ. Outdoor Learn..

[35] Badau A., Rachita A., Sasu C.R., Clipa A. (2018). Motivations and the Level of Practicing Physical Activities by Physio-Kinetotherapy Students. Educ. Sci..

[36] Cañabate D., Martínez G., Rodríguez D., Colomer J. (2018). Analysing Emotions and Social Skills in Physical Education. Sustainability.

[37] Badau D., Badau A. (2018). The motor, educational, recreational and satisfaction impact of adventure education activities in the urban tourism environment. Sustainability.

[38] San Pedro Veledo M.B., López Manrique I., Fombella Coto I., del Cura González Y., Sánchez Martínez B., Álvarez González A.I. (2018). Social Sciences, Art and Physical Activity in Leisure Environments. An Inter-Disciplinary Project for Teacher Training. Sustainability.

[39] Amado-Alonso D., Mendo-Lázaro S., León-del-Barco B., Mirabel-Alviz M., Iglesias-Gallego D. (2018). Multidimensional Self-Concept in Elementary Education: Sport Practice and Gender. Sustainability.

[40] Şahin E., Çekin R., Yazıcılar Özçelik İ. (2018). Predictors of Academic Achievement among Physical Education and Sports Undergraduate Students. Sports.

[41] Brito R.M., Rodríguez C., Aparicio J.L. (2018). Sustainability in Teaching: An Evaluation of University Teachers and Students. Sustainability.

[42] Martins L.V., Tezza R., Dias Alperstedt G., Campos M.S.L. (2017). Future Professionals: A Study of Sustainable Behavior. Sustainability.

[43] Lisinskiene A. (2018). The Effect of a 6-Month Coach Educational Program on Strengthening Coach-Athlete Interpersonal Relationships in Individual Youth Sport. Sports.

[44] Donnelly J.E., Hillman C.H., Castelli D., Etnier J.L., Lee S., Tomporowski P., Lambourne K., Szabo-Reed A.N. (2016). Physical activity, fitness, cognitive function, and academic achievement in children: A systematic review. Med. Sci. Sports Exerc..

[45] Dobbins M., Husson H., DeCorby K., LaRocca R.L. (2009). School-based physical activity programs for promoting physical activity and fitness in children and adolescents aged 6 to 18. Cochrane Libr..

[46] Healy G.N., Wijndaele K., Dunstan D.W., Shaw J.E., Salmon J., Zimmet P.Z., Owen N. (2008). Objectively measured sedentary time, physical activity, and metabolic risk: The Australian Diabetes, Obesity and Lifestyle Study (AusDiab). Diabetes Care.

[47] Matthews C.E., Chen K.Y., Freedson P.S., Buchowski M.S., Beech B.M., Pate R.R., Troiano R.P. (2008). Amount of time spent in sedentary behaviors in the United States, 2003–2004. Am. J. Epidemiol..

[48] Felez-Nobrega M., Hillman C.H., Dowd K.P., Cirera E., Puig-Ribera A. (2018). ActivPALTM determined sedentary behaviour, physical activity and academic achievement in college students. J. Sports Sci..

[49] Kilpatrick M., Hebert E., Bartholomew J. (2005). College Student’s motivatin for physical activity. J. Am. Coll. Health.

[50] Lee S.Y., Sung H.K. (2016). Motivation for Participation in Winter Sports. Sport Inf. Technol. Res..

[51] Špehar N., Gošnik J., Reichel K.F. The preferences toward sports of students in institutions of higher education. Proceedings of the 5th International Scientific Conference on Kinesiology.

[52] Mraković S., Hraski M., Lorger M. Differences in preferences toward sport activites of female students on university of Zagreb. Proceedings of the 6th International Scientific Conference “Integrative Power of Kinesiology”.

